# The effect of taxation on sustainable development goals: evidence from emerging countries

**DOI:** 10.1016/j.heliyon.2022.e10512

**Published:** 2022-09-02

**Authors:** Md. Mominur Rahman

**Affiliations:** a Department of Accounting and Information Systems, Comilla University, Shalmanpur, Bangladesh; b Department of Business Administration, BGIFT Institute of Science and Technology (BIST), Gazipur, Bangladesh

**Keywords:** Corporate tax rate, Sustainable development, SDG index, BRIC, CIVETS

## Abstract

The purpose of the study is to examine the effects of the corporate tax rate on sustainable development in the BRIC and CIVETS countries. This research employs a panel dataset for 2000–2021 years and applies panel data regression model to analyse the data. The study confirms the results checking the robustness through the fully modified ordinary least square and the dynamic ordinary least square panel estimate methods. The study passes several tests like cross-sectional dependence tests, unit root tests, and model selection tests before conducting the focal part of the analysis. The research finds that the corporate tax rate is positively and significantly associated with the sustainable development goals (SDG). The result implies that a higher rate of corporate tax plays vital role in achieving the sustainable development goals in the emerging economies. By including personal income tax, sales tax, and theoretical arguments, the study contributes to the debate on the corporate tax rate and the achievement of SDG in the emerging countries. The study applies both individual effects and combined effects of corporate tax rate, personal income tax, sales tax, and effective tax rate with SDG. In both cases, the research finds significant and positive association of taxation with SDG. Thus, the study argues that achieveing the SDG of emerging economies depends on the countries' taxation rate and policy. This research employs the most updated data set that also contributes to the existing literature of emerging economies. Thus, the findings generated from this study can be a policy dialogue for the academics, policy-makers and government bodies of BRIC and CIVETS countries and other emerging economies as well.

## 1 Introduction

The global average statutory corporate income tax rate is 23.54 percent, based on data from 180 jurisdictions. Based on GDP, the average statutory tax rate is 25.44 percent. A regional average of 19.62 percent is the lowest in Asia and 27.97 percent in Africa^1^. The effects of changes in corporate income taxation on economic growth have sparked controversy in academic and policy circles. Lower tax rates, according to proponents of corporate tax cuts, would increase growth. Others have questioned whether corporate tax cuts will have a significant positive influence on growth. Indeed, throughout the last four decades, corporate tax rates have fallen, and corporate tax bases have grown worldwide, but to varying degrees in different nations (Asen, 2020; Heimberger, 2021).

Even the world's most powerful economy, located in the centre of the globe, has seen its economy go into decline. The statutory corporate income tax rate in the United States was cut from one of the highest in the world by the 2017 tax reform. Countries and tax jurisdictions that have been examined include the United States, which has a corporate income tax rate that was the fourth highest worldwide in 2017 (Jahnsen and Pomerleau, 2017). Europe has lower business income tax rates than many other regions, although many emerging countries have higher corporation tax rates than the world average (Asen, 2020).

Economic growth has long been a contentious issue in political and economic circles. To match this, several research studies on this topic in general and the effects of taxes, in particular, have been conducted. Unfortunately, the goal of these studies has frequently been political, whether as a campaign tool for a new presidential candidate or as an empirical conclusion to support congress' repeal/enactment of a bill. In reality, among other things, economic performance is the most crucial criterion for evaluating a politician's stay in office. This should unquestionably be the case for obvious reasons (Romer and Romer, 2010).

Economic growth is a long-term increase in an economy's productive potential. This helps to clarify a crucial point: the distinction between long-term economic development and the short-term economic changes marked by booms and busts that occur every few months or years, known as business cycles. Because it is a typical social activity, there are few concrete explanations or accurate periodic estimations for growth rate. There has been a wide range of yearly economic growth in countries, and much remains unclear about how complex sociological, economic, and political forces interact to produce economic growth (Mutén and Faxén, 1966). According to Solow (1970), growth is dependent on the accumulation of labor and capital. As a result, empirical research on the effects of taxation on labor and capital takes these aspects into account (Solow, 1970). Lee and Gordon (2005) agreed, claiming that excluding positive externalities from the neoclassical model provides more explanation for growth. They go on to say that these externalities are generated by research & development and entrepreneurial activity (Lee and Gordon, 2005). Equipment investment can have significant beneficial spillovers, according to De-Long and Summers (1991). Evans and Rauch (1999) added (Webberian) bureaucracy to the list of growth factors.

The purpose of taxation is to produce enough revenue to keep the government running while causing the least amount of harm to the economy. A more realistic reality requires the government to use tax policy as a steering wheel to impose economic control. The risk-taking is inhibited by a progressive tax system (Gentry and Hubbard, 2000). Gordon (1998) argues that incorporation encourages risk-taking by decreasing company tax rates in comparison to personal tax rates. Gordon and Cullen (2002) employs tax return data from 1964 to 1993 to examine the many possible consequences of the tax system on entrepreneurship. Tax impacts were shown to have substantial empirical evidence. A great method for tracking the rate of economic expansion is to look at the effect taxes have on GDP.

However, a negative relationship between company tax rates and GDP is predicted by economic theory (Devereux et al., 2008; Kawano and Slemrod, 2016). In other words, the corporate tax multiplier impact has been found to be negative. Deductions from income reduce the reported earnings of the firm and are thus regarded an extra expense. Taxes are in direct conflict with a company's primary goal of reducing expenses while boosting revenue. Except in a few rare cases, it must be done regardless of the expense.

The study aims to examine the effects of the corporate tax rate on sustainable development in the BRIC and CIVETS countries. This study tries to contribute to BRIC and CIVETS countries by the following questions:

1) What role does corporate taxation play in promoting long-term economic development in BRIC and CIVETS countries?
2) What role does personal taxation play in boosting long-term economic development of BRIC and CIVETS economies?
3) What role does sales tax play in the long-term economic development of the BRIC and CIVETS economies?
4) What impact does the effective tax rate have on the BRIC and CIVETS economies' long-term economic development?
5) What effect does political stability have on the long-term economic development of the BRIC and CIVETS economies?

To investigate these answers, this research employed various current panel econometric approaches, including CSD test following Breush-Pagan LM, Pesaran scaled LM, and Pesaran CD; the Levin-Lin-Chu unit root test (LLC), the Im, Pesaran, and Shin (IPS) unit root test, and the Breitung unit root test for investigating the stationary of the series applying a variety of unit root tests as De-Hoyos and Sarafidis (2006), Breitung and Das (2005), and Sarafidis and Wansbeek (2012). Further, the study applied the Chow and Breush-Pagan tests to decide whether pooled OLS (Ordinary Least Square) or the fixed and random effects model suggested by Pindado and Requejo (2015). Thus, the study employed the fully modified ordinary least square (FMOLS) and the dynamic ordinary least square (DOLS) panel estimate methods to check the robustness of the main findings as Rahman et al. (2021), Lu and White (2014), and Frehse et al. (2009). The findings revealed that the corporate tax rate (CTR) is positively and significantly related to the sustainable development goal (SDG) using a random effect. It suggests that a higher corporate tax rate promotes the SDG that helps achieve sustainable development goals in BRIC and CIVETS countries.

The structure of the paper includes section one: introduction, section two: literature review, section three: methodology, section four: results and discussions, section five: Robustness of results, and section six: conclusion, policy implications, and future research scope.

## 2 Literature review

### 2.1 Theoretical literature

The scope of this essay does not provide an in-depth discussion of the theoretical literature on the link between corporation tax and economic development. This section provides an overview of key theoretical concepts that have guided and informed much of the relevant empirical research.

Economic growth is dependent on the accumulation of the production variables labour and capital in the neoclassical context of the Solow (1970) model. Any given tax system creates a capital-labour ratio equilibrium, and exogenous technical development causes a further rise in GDP per capita. There should be no long-term influence of tax on economic growth, regardless of the extent of potential tax structure misallocations (Heimberger, 2021; Martínez-Ferrero and Frías-Aceituno, 2015). Growth and the amount of per capita income in a steady state can be affected negatively by capital income taxes, and the shift between equilibrium levels might take years or decades. Numerous decisions made by individuals in the economy (such as schooling or R&D investment) have recently been connected to aggregate economic development under the notion of endogenous growth, and these decisions can be impacted by financial regulation (Aghion and Howitt, 2008; Solow, 1970).

Corporations' ability to diminish tax incentives is one of several theoretical issues that have prompted hypothesis testing in empirical research (Ferede and Dahlby, 2012; Shevlin et al., 2019). Raising taxes lowers after-tax income because capital costs rise. Economic growth may be stifled by higher taxes, which may prevent investment (innovative activities). Total Factor Productivity (TFP) can also be affected by corporation taxes, which distort factor pricing and lead to inefficient resource allocation. As a result of decreased efficiency, TFP may be lowered. Furthermore, greater taxes may have a negative impact on entrepreneurial activities, reducing TFP (Djankov et al., 2010).

The optimal corporation tax rate isn't zero, and higher taxes can actually enhance economic development in some cases, according to newer models of tax policy (Atkeson et al., 1999). Aghion et al. (2013) show that capital taxes can increase economic growth by moving the tax burden away from labour taxation using an innovation-based growth model. Jones et al. (1993) show that capital taxation can stimulate growth if tax revenues are used to fund more productive government spending. Tax cuts that "starve the beast" may, in turn, lead to a reduction in the provision of productive public capital (Fuest et al., 2019). Corporate income taxation may stimulate economic growth, while asset income taxation may restrain it in its upward trajectory. Corporations' investment and location incentives can be influenced by governments' control over corporate tax rates and the tax base (Peretto, 2003).

Measuring tax increases on corporations is critical to determining their effect on economic growth (Devereux et al., 2008). When it comes to determining where to set up shop, effective average tax rates are more relevant than statutory tax rates because they take into account both rate and base changes. Effective marginal tax rates are also significant because they play a role in the decision-making process regarding incremental investments (Gentry and Hubbard, 2000; Lee and Gordon, 2005).

### 2.2 Empirical literature

Lee and Gordon (2005) employs cross-sectional data from 1970 to 1997 for a group of 70 advanced and developing countries to analyse how tax policy influences economic growth. Short-term impacts may be smoothed out by looking at the link between (company) tax rates and long-term development. OLS is their favourite estimating approach, yet corporation tax rates may be endogenous to economic growth (Lee and Gordon, 2005). According to Lee and Gordon (2005), cross-sectional disparities in economic growth rates may be explained by statutory corporation tax rates.

According to Arnold et al. (2011), raising taxes on corporations has a greater negative impact on economic growth than doing the same for individuals. Data from 21 OECD countries from 1971 to 2004 was utilised in a dynamic panel data model and a Pooled Mean Group estimator by Arnold et al. (2011). Accordingly, they divide total tax revenue by the total tax revenue collected to arrive make impact corporate taxes. Corporate income taxes as a proportion of overall taxation have a positive, though inconsistent, association with economic growth (Widmalm, 2001). Angelopoulos et al. (2007) estimate panel data models for 23 OECD countries from 1970 to 2000 and comes to similar findings. According to Arnold et al. (2011), corporation tax modifications should be considered in connection with changes in other tax components or government spending owing to budget constraints. If overall tax revenues are corrected for in the underlying regression, a corporate tax decrease is expected to be offset by tax increases in other categories. When government expenditure is considered, a company tax decrease is unlikely to result in changes in spending (Arnold et al., 2011). According to Kate and Milionis (2019), Higher company taxes may boost growth in technologically advanced industrialised nations by stimulating private innovation and providing resources for constructive governmental investment. Lowering tax wedges may result in a more negative association between corporate tax rates and growth in trailing nations that are more focused on technology imitation (Kate and Milionis, 2019).

The identification of growth impacts of company taxes may be less tainted by institutional or geographical factors when data variation between states within the same nation is used. They reach a range of conclusions about the impact of corporate taxes on economic development (Kate and Milionis, 2019). Alm and Rogers (2011) employ data from 48 US states from 1959 to 1997 to examine the link between company taxes and state economic development. They discover evidence of a link between corporate taxes and state economic development. Panel data for 50 US states from 1977 to 2005 and a broad set of tax, expenditure, and political control variables are used by Prillaman and Meier (2014). They found that there has no impact on state economic growth.

## 3 Methodology

In this section, the study explains the data, data coverage period, variables and their sources, and econometric model. The study aims to examine the effects of the corporate tax rate on sustainable development in the BRIC and CIVETS countries.

### 3.1 Data and variables

The study collected quantitative data from a balanced panel of ten emerging countries (BRIC: Brazil, Russia, India and China, and CIVETS: Colombia, Indonesia, Vietnam, Egypt, Turkey, and South Africa) for 22 years (2000–2021) and 220 is the number of observations. The study uses STATA 16 to conduct the analysis, and the variables employed in this research are presented in Table 1. The dataset pertaining to the BRIC and CIVETS countries was compiled to conduct empirical research, as these countries are mostly regarded as emerging economies. According to OECD^2^, in promoting sustainable development, taxation plays a dominant role in the emerging countries because emerging nations face substantial challenges to formulate tax capacities and mobilise domestic resources. As this study proposes the association between SDGs and taxation, BRIC and CIVETS will be appropriate. All of the information is gathered from secondary sources that are publicly available. Table 1 contains information about the data sources, including their descriptions, units, and sources of information.

**Table 1:** Descriptions of the variables.

Variables	Sign	Measurement	Sources
**Dependent Variable**			
Sustainable Development Goals	SDG	SDG indicates sustainable development goals (SDG Index) of each country at each year.	(Diaz-Sarachaga, 2018, Sachs, 2021, Walker, 2001)
***Independent Variable***			
Corporate Tax Rate	CTR	CTR indicates the average corporate tax rate of each country at each year.	(Djankov et al., 2010; Kawano and Slemrod, 2016; Prillaman and Meier, 2014)
Personal Income Tax Rate	PIT	PIT indicates personal income tax rate of each country at each year.	(Kawano and Slemrod, 2016; Walker, 2001)
Sales Tax Rate	STR	STR indicates sales tax rate of each country at each year.	(Jahnsen and Pomerleau, 2017; Lee and Gordon, 2005)
Effective Tax Rate	ETR	ETR indicates composite effective average tax rate of each country at each year	(Ferede and Dahlby, 2012; Heimberger, 2021; Widmalm, 2001)
***Macroeconomic Variable***			
GDP Growth	GR	GDP growth rate of each country at each year.	(Hussain et al., 2021; Rahman et al., 2021; Romer and Romer, 2010)
Inflation	INF	The inflation rate of each country at each year	
***Country-level Variable***			
Financial Development	FD	The financial development index is a broad measure for financial development by taking into account its efficiency, accessibility, and depth. It takes value from each country at each year.	(Busch et al., 2015; Rahman et al., 2021; Shevlin et al., 2019)
Financial Openness	FOP	KAOPEN (The Chinn-Ito Index) index measures constraints on capital and current account transactions, the requirement for the surrender of export proceeds, and the presence of multiple exchange rates. The index ranges from 0 to 1, where a higher value indicates more financial openness	(Hussain et al., 2021; Rahman et al., 2021; Romer and Romer, 2010)
Political Stability	PSI	The political stability index reflects the possibility of politically-motivated violence, including terrorism. Variable ranges from −2.5 to 2.5, with higher values indicating more political stability	(Rahman et al., 2021; Yakubu et al., 2021)

Source: Developed by the authors.

Sustainable development goal (SDG) is the main dependent variable that is measured by the annual SDG index of each country. The higher and positive values of SDG indicate the better achievement of sustainable development goals. SDG data has been collected from the SDG Index. The tax rate is the main independent variable that is proxied by corporate tax rate (CTR), personal income tax rate (PIT), sales tax rate (STR), and effective tax rate (ETR). Tax is intrinsically linked to development as taxation provides the revenue that states need to mobilize resources and reinforce acountry's infrastructure. Taxation plays an essential role in achieving the SDGs. Taxes are required for governments to make the necessary environmental and social investments (Jahnsen and Pomerleau, 2017). Tax plays an important role in collecting revenues for the government. Thus, the higher rates of CTR, PIT, STR, and ETR, the better the roles of taxation. Healthy collections of tax provide state and local governments the money to improve education, hire police officers and firefighters, build and maintain roads, keep parks clean, and many other public services. Tax rate data has been collected from Tax Foundation (CTR), Trading Economics (PIT and STR), and OECD Statistics (ETR). The study employs GDP growth rate and inflation as macro-economic variables, financial development, financial openness data collected from, and political stability data collected as country-level control variables to control the relationship of sustainable development goals and tax rate (see Table 1). The data of the control variables are collected from WDI (GDP growth and inflation), IMF (financial development), KNOEMA (financial openness), and Global Economy (political stability data).

### 3.2 Model specification

In this study, our main objective is to examine the role of taxation in achieving sustainable development goals; essentially, this study specifies the following empirical model (Equation 1):

(1) $$SDG_{it}=C+\beta_{1}Tax\;Rate_{it}+\overset{C}{\underset{c=1}{\sum }}\beta_{c}Y_{it}^{c}+\;\in_{it}$$

where i and t subscripts stand for the country and year, respectively. This study uses the sustainable development goal (SDG) index as the dependent variable in different specifications. The tax rate is the independent variable that is proxied by CTR (corporate tax rate), PIT (personal income tax rate), STR (sales tax rate), and ETR (effective tax rate). C is a constant term. Y _it_ with superscripts c are the vectors of control variables, and ∈_it_ is the error term. Detailed definitions and data sources of the variables are presented in Table 1.

This paper uses four measures of tax rate and re-writes Eq. (1) as follows (see Eqs. 1a, 1b, 1c and 1d):

(1a) $$SDG_{it}=C+\lambda \;CTR_{it}+\overset{C}{\underset{c=1}{\sum }}\beta_{c}Y_{it}^{c}+\in_{it}$$

Here, CTR is the corporate tax rate.

(1b) $$SDG_{it}=C+\lambda \;PIT_{it}+\overset{C}{\underset{c=1}{\sum }}\beta_{c}Y_{it}^{c}+\in_{it}$$

Here, PIT is the personal income tax rate.

(1c) $$SDG_{it}=C+\lambda \;STR_{it}+\overset{C}{\underset{c=1}{\sum }}\beta_{c}Y_{it}^{c}+\in_{it}$$

Here, STR is the sales tax rate.

(1d) $$SDG_{it}=C+\lambda \;ETR_{it}+\overset{C}{\underset{c=1}{\sum }}\beta_{c}Y_{it}^{c}+\in_{it}$$

Here, ETR is the effective tax rate.

In this case, the researchers employed combined effects of all the independent variables as follows in Eq. (2):

(2) $$SDG_{it}=C+\beta_{1}CTR_{it}+\beta_{2}PIT_{it}+\beta_{3}STR_{it}+\beta_{4}ETR_{it}+\overset{C}{\underset{c=1}{\sum }}\beta_{c}Y_{it}^{c}+\;\in_{it}$$

The study runs several tests and steps to select the best-fitted estimation methods (Breitung and Das, 2005; De-Hoyos and Sarafidis, 2006; Hadri and Kurozumi, 2012; Hussain et al., 2021; Lu and White, 2014). First, the cross-sectional dependence (CSD) test has been performed as CSD is one of the most important diagnostics that a researcher should investigate before performing a panel data analysis. Second, a unit root test has been run to confirm the absence of spurious relations amongst the variables. Third, a test to select a panel regression model has been performed. Finally, a robustness check with an alternative estimation method has been performed.

## 4 Results and discussions

### 4.1 Summary statistics and correlation matrix

Summary statistics of the variables and the correlation matrix, have been presented in Table 2. Regarding the SDG, the overall mean is 0.67 with a standard deviation of 0.19. The moderate level of average SDG values indicate that BRIC and CIVETS countries are more likely to achieve sustainable development goals. The mean of taxation proxies CTR, PIT, STR, and ETR are 0.62, 0.30, 0.38, and 0.41 with S.D. of 0.26, 0.17, 0.11, and 0.06, respectively. Moreover, Table 2 also represents the pairwise Pearson correlations between employed variables. The correlation between the independent variables is less than 80% proves that the issue of multicollinearity is not undermining our findings (Yakubu et al., 2021).

**Table 2:** Summary statistics and correlation matrix.

Criteria	SDG	CTR	PIT	STR	ETR	GR	INF	FD	FOP	PSI
Mean	0.67	0.62	0.30	0.38	0.41	0.58	0.16	0.49	0.29	0.31
Median	0.63	0.56	0.24	0.35	0.37	0.51	0.13	0.42	0.23	0.28
Maximum	0.78	0.64	0.44	0.46	0.53	0.67	0.32	0.51	0.42	0.32
Minimum	0.42	0.46	0.32	0.23	0.27	0.31	0.18	0.22	0.31	0.23
SD	0.19	0.26	0.17	0.11	0.06	0.13	0.03	0.08	0.16	0.13
Skewness	1.87	1.28	−0.82	1.16	1.29	−0.29	2.11	1.18	0.73	1.11
Kurtosis	2.19	4.21	2.78	3.52	2.17	3.11	2.38	2.22	3.73	3.12
Obs.	220	220	220	220	220	220	220	220	220	220
**Variables**		**CTR**	**PIT**	**STR**	**ETR**	**GR**	**INF**	**FD**	**FOP**	**PSI**
CTR		1								
PIT		.329∗	1							
STR		.578∗	.582∗	1						
ETR		.266∗	.285∗	.450∗	1					
GR		.237∗	.235∗	.394∗	.641∗	1				
INF		.321∗	.355∗	.515∗	.474∗	.564∗	1			
FD		.240∗	.258∗	.236∗	.249∗	.293∗	.235∗	1		
FOP		.156∗	.181∗	.228∗	.187∗	.381∗	.337∗	.667∗	1	
PSI		.288∗	.307∗	.441∗	.528∗	.456∗	.482∗	.312∗	.290∗	1

∗ indicates 1% level of significance.

Source: The authors developed

It was expected that there would be no data disturbance due to cross-sectional dependency while analyzing panel data. However, as economies are interconnected mostly at regional and global levels, cross-sectional dependency is frequent in panel data (De-Hoyos and Sarafidis, 2006). Table 3 displays the CSD (Cross-sectional dependence) test following Breush-Pagan LM, Pesaran scaled LM, and Pesaran CD criteria. No cross-sectional dependency was the null hypothesis that was rejected 1%, 5%, and 10% level of significance. Thus, the study finds the existence of cross-sectional dependency among the cross-section units. After the CSD test, the study investigated whether the series are stationary or non-stationary in the next subheading.

**Table 3:** Cross-sectional dependence test.

Variables	Breusch-Pagan LM	Pesaran scaled LM	Pesaran CD
SDG	−9.495∗∗	−9.500∗∗	−9.516∗
CTR	−12.418∗	−12.390∗∗	−12.446∗∗
PIT	−9.919∗∗	−9.890∗	−9.943∗∗
STR	−10.436∗	−10.420∗∗	−10.461∗∗
ETR	−12.574∗∗	−12.560∗∗	−12.603∗
GR	−11.840∗	−11.810∗∗	−11.868∗
INF	−10.106∗	−10.080∗∗	−10.125∗
FD	−10.224∗∗	−10.200∗∗	−10.248∗
FOP	−9.215∗∗∗	−9.190∗∗	−9.231∗∗
PSI	−9.495∗∗∗	−9.500∗∗	−9.516∗∗∗

∗∗∗, ∗∗, ∗ indicates 1%, 5%, and 10% level of significance, respectively.

Source: Authors developed

### 4.2 Stationary test

The stationarity of series, particularly when it comes to financial or macroeconomic data, is a necessity for any experimental and especially econometric research before moving on to econometric interpretation (Hadri and Kurozumi, 2012; Pindado and Requejo, 2015). Stationary indicates that variance, mean, and autocorrelation framework of the statistical series are constant over time but these properties change over time if the series are non-stationary. Thus, the study investigated the stationary of the series applying a variety of unit root tests including the Levin-Lin-Chu unit root test (LLC), the Im, Pesaran, and Shin (IPS) unit root test, and the Breitung unit root test (Hadri and Kurozumi, 2012). Table 4 shows the results of these three tests. The null hypothesis (The series are non-stationary) of the tests was rejected at 1%, 5%, and 10% level of significance revealing that the series is stationary (Breitung and Das, 2005). The findings concluded that the series had unit roots, but after first differencing, they become stationary (see the decisions in Table 4). As the data are stationary, the study can run the fitted regression model to examine the association between sustainable development goals and taxation.

**Table 4:** Test of unit root.

Variables	Breitung Test		LLC Test		IPS Test		Decisions
	C	C + T	C	C + T	C	C + T	
ΔSDG	−14.29∗∗	−14.27∗∗	−14.29∗	14.27∗∗	9.49∗∗	9.50∗∗	I (1)
ΔCTR	13.94∗∗	−13.92∗∗	13.94∗∗	13.92∗∗∗	12.42∗∗∗	12.39∗∗	I (1)
ΔPIT	13.50∗∗∗	−13.49∗	13.50∗∗	−13.49∗∗	9.92∗∗	−9.89∗∗∗	I (1)
ΔSTR	−13.96∗	13.93∗∗	13.96∗∗	13.93∗∗	10.44∗∗∗	10.42∗∗	I (1)
ΔETR	−13.39∗∗	13.40∗∗	−13.39∗∗	−13.40∗∗	12.57∗∗	−12.56∗∗∗	I (1)
ΔGR	13.71∗	13.78∗	−13.71∗∗∗	13.78∗∗∗	−11.84∗∗	11.81∗∗	I (1)
ΔINF	13.73∗	−14.10∗∗∗	13.73∗∗	14.10∗∗	10.11∗∗	10.08∗	I (1)
ΔFD	−14.19∗∗	−14.67∗∗	−14.19∗∗∗	14.67∗∗	−10.22∗∗∗	10.20∗	I (1)
ΔFOP	14.85∗∗	14.89∗	14.85∗∗	14.89∗∗∗	9.22∗∗	−9.19∗∗	I (1)
ΔPSI	14.29∗∗∗	14.27∗∗∗	14.29∗∗∗	−14.27∗∗∗	9.49∗∗∗	9.50∗∗∗	I (1)

∗∗∗, ∗∗, ∗ indicates 1%, 5%, and 10% level of significance, respectively. C=Constant and T = Trend.

Source: Authors developed

### 4.3 Model selection

The study found that the series are cross-sectional dependent but stationary after first differencing (see Tables 3 and 4). The study applied the Chow and Breush-Pagan tests to decide whether pooled OLS (Ordinary Least Square) or fixed and random effects model as Pindado and Requejo (2015) suggested. The null hypothesis (pooled OLS is an effective model) of both tests was rejected at 1% and 5% levels of significance. The findings revealed that fixed and random effects models are effective to examine the relationship between global entrepreneurship development and trade openness (see Table 5).

**Table 5:** Panel regression selection criteria.

Test	p-value	Decision
Chow (F-test)	0.034∗∗	Reject null hypothesis (pooled OLS is effective model)
BP (χ^2^ test)	0.013∗∗∗	Reject null hypothesis (pooled OLS is effective model)

∗∗∗, ∗∗ indicates 1% and 5% level of significance, respectively.

Source: Authors constructed

The results of the Chow test required us to employ the fixed effects model, but the results of the BP test required us to use the random-effects model (Pindado and Requejo, 2015). As a result, the Hausman test was used to choose which model to use: random effects or fixed effects (Amini et al., 2012). The null hypothesis (Random-effects model should be used) of the Hausman test was accepted indicating that the random-effects model needs to be employed (see Table 6). As the fitted model has been chosen, the study investigated the impact of sustainable development goals on the tax rate in the next subheading.

**Table 6:** Results of Hausman test.

Test	p-value	Decision
Cross-section random	0.368	Accept null hypothesis (Use Random-effects model)
Period random	0.257	Accept null hypothesis (Use Random-effects model)
Cross-section and period random	0.486	Accept null hypothesis (Use Random-effects model)

Source: Authors constructed

### 4.4 Regression outputs

Table 7 represents the regression output based on the random-effects model, and different algorithms and measures of model fitness have been reported. The findings revealed that corporate tax rate (CTR) is positively and significantly related to SDG indicating that a 1% increase in CTR increases 0.122% of SDG individually, and 0.019% of SDG combinedly. The finding suggests that a higher corporate tax rate promotes the SDG index that helps achieve sustainable development goals in BRIC and CIVETS countries. Corporate tax policies in BRIC and CIVETS countries bring about the necessary condition for economic progress, job creation, and poverty reduction. Thus, while no cure exists, it is clear that closer convergence of investment and tax policy is necessary to promote investment, job creation, and economic growth. International business continues to be a potent instrument for assisting people in escaping poverty (Arnold et al., 2011). Taxation is inextricably related to development since it generates the funds required for states to mobilize resources and strengthen a country's infrastructure particularly in the emerging economies like BRIC and CIVETS. Thus, the emerging countries can focus on the corporate tax rate advancement for the sustainable development that is somehow consistent with the study of Busch et al. (2015) and Heimberger (2021).

**Table 7:** Results of regression estimation (Random-effects model).

Variables	Individual Effects				Combined Effects (SDG)
	SDG	SDG	SDG	SDG	
Constant	0.018∗∗∗	0.036∗∗∗	0.041∗∗∗	0.026∗∗∗	0.105∗∗∗
Δ**CTR**	**0.122∗∗**				**0.019∗∗**
Δ**PIT**		**0.009∗**			**0.028∗**
Δ**STR**			**0.114∗∗**		**0.196∗∗**
Δ**ETR**				**0.219∗∗∗**	**0.032∗∗∗**
ΔGR	0.197∗	0.202∗	0.196∗	0.183∗	0.201∗
ΔINF	−0.205	−0.200	−0.228∗	−0.165	−0.163
ΔFD	0.319∗∗	0.318∗∗	0.340∗∗∗	0.325∗∗	0.321∗∗∗
ΔFOP	0.179∗	0.177∗∗	0.171∗∗	0.177∗	0.175∗∗
ΔPSI	0.262∗∗∗	0.261∗∗∗	0.270∗∗∗	0.245∗∗	0.249∗∗
R-square	0.4786	0.4382	0.4740	0.4846	0.4853
Adj. R-square	0.4639	0.4231	0.4592	0.4701	0.4633
SE of regression	2.8684	2.8257	2.8810	2.8518	2.8701
F-statistic	32.5810∗∗∗	32.1827∗∗∗	31.9864∗∗∗	33.3793∗∗∗	22.0012∗∗∗
Durbin–Watson statistic	2.1675	2.1777	2.1851	2.1889	2.1823
Diagnostic tests					
Wooldridge autocorrelation test p value	0.171	0.308	0.218	0.176	0.438
Greene heteroscedasticity test p value	0.317	0.467	0.329	0.409	0.412

Note: The null hypothesis (Absence of autocorrelation) of the Wooldridge autocorrelation test was accepted and the null hypothesis (error terms are normally distributed) of the Greene heteroscedasticity test was accepted. ∗∗∗, ∗∗, ∗ indicates 1%, 5%, and 10% level of significance, respectively.

Source: Authors constructed

Similarly, our findings regarding personal income tax rate (PIT), sales tax rate (STR), and effective tax rate (ETR) imply a positive and significant relationship with SDG by individually and combinedly, demonstrating that 0.009% of SDG individually, 0.028% of SDG combinedly can be improved through 1% increase in PIT; 0.114% of SDG individually, 0.196% of SDG combinedly can be upgraded through 1% increase in STR; and 0.219% of SDG individually, 0.032% of SDG combinedly can be enhanced through 1% increase in ETR in BRIC and CIVETS countries. Thus, tax at individual assessments, business assessments, and control of tax avoidance are the cruicial factors that are associated with country's sustainable development. In emerging countries, the businesses are incorporating the SDGs into their company strategy and fundamental operations (Shevlin et al., 2019). This also implies that the organization's tax strategy should emphasize its contribution to the SDGs, particularly the emerging nations. The roles of taxation is to contribute sustainable development that is consistent with the findings of Ferede and Dahlby (2012) who argued that taxes are the required determinant of sustainable economic development.

The study found positive associations of GDP growth (GR), financial development (FD), financial openness (FOP), and political stability (PSI) with sustainable development goals. These findings are consistent with the study of Bekhet and Othman (2018); Busch et al. (2015); Hussain et al. (2021); Martínez-Ferrero and Frías-Aceituno (2015), and Shahbaz et al. (2016). The findings indicated that the higher the GDP growth, financial development, financial openness, and political stability in BRIC and CIVETS nations, the more the possibility of an improved SDG index. Thus, as the emerging nations, BRIC and CIVETS can focus on improving GR, FD, FOP, and PSI to enhance sustainable development goals. Results revealed that inflation is negatively related to SDG but it was not significant (Only one model shows significance at 10% level)). However, inflation may hamper sustainable development goals as the study found negative relations. Thus, the countries money market should be controlled effectively.

## 5 Robustness of results

### 5.1 Robustness check: cross-group analysis

The study investigated that taxation significantly influences sustainable development goals in the emerging nations i.e., BRIC and CIVETS all samples considered (see Table 7). Now, the authors investigated whether the found relationships vary at cross-group analysis i.e., BRIC nations and CIVETS nations as Davidov et al. (2012) and Maddala (1999) aurged. In Table 8, the study displayed the group-wise regression results that imply cross-country-group relations between taxation and sustainable development goals that is similar to Bansal et al. (2021). More specifically, this research emphasized finding the relations in specific group-country separately. The study found, in this case, that taxation significantly influences sustainable development goals in BRIC and CIVETS economies. CTR, PIT, STR, and ETR upgrade SDG in BRIC and CIVETS countries that are consistent with the results of Table 7.

**Table 8:** Results of regression estimation (Random-effects model).

Country		Individual Effects				Combined Effects (SDG)
		SDG	SDG	SDG	SDG	
BRIC	Constant	0.187∗∗	0.132∗∗	0.003∗∗	0.110∗∗	0.322∗∗
	Δ**CTR**	**0.131∗∗**				**0.109∗∗**
	Δ**PIT**		**0.108∗**			**0.105∗**
	Δ**STR**			**0.224∗∗**		**0.243∗**
	Δ**ETR**				**0.193∗∗**	**0.089∗∗**
	Control Variables	Yes	Yes	Yes	Yes	Yes
	R-square	0.6912	0.7327	0.6263	0.5728	0.5317
	F-statistic	78.611∗∗∗	76.322∗∗∗	84.138∗∗∗	65.321∗∗∗	46.176∗∗∗
CIVETS	Constant	0.010∗∗	0.226∗∗	0.314∗∗	0.043∗∗	0.347∗∗
	Δ**CTR**	**0.095∗∗**				**0.209∗∗∗**
	Δ**PIT**		**0.110∗∗**			**0.163∗∗**
	Δ**STR**			**0.141∗∗**		**0.012∗**
	Δ**ETR**				**0.213∗∗∗**	**0.027∗∗∗**
	Control Variables	Yes	Yes	Yes	Yes	Yes
	R-square	0.4281	0.4336	0.4369	0.4289	0.5267
	F-statistic	149.378∗∗∗	157.697∗∗∗	134.182∗∗∗	148.089∗∗∗	152.743∗∗∗

∗∗∗, ∗∗, ∗ indicates 1%, 5%, and 10% level of significance, respectively.

Source: Authors constructed

### 5.2 Robustness check: alternative estimation methods (FMOLS and DOLS)

The study checked the robustness of the findings. Researchers of different academics suggested checking robustness with alternative estimation methods (Lu and White, 2014; Mahmood et al., 2020; Rahman et al., 2021; Rojas-Vallejos and Lastuka, 2020). The fully modified ordinary least square (FMOLS) and the dynamic ordinary least square (DOLS) are the methods that produce reliable estimations if the small size is small and provide a check for the robustness of the findings. The FMOLS and DOLS can deal with serial correlation. Thus, the study employed the FMOLS and the DOLS panel estimate methods to check the robustness of the main findings. The findings of FMOLS (Column 2–6) and DOLS (Column 7–11) are displayed in Table 9. The study revealed that CTR, PIT, STR, and ETR are positively significant to affect SDG in both FMOLS and DOLS models. The findings of FMOLS and DOLS are consistent with the main findings presented in Table 7 (see Table 9 for robustness checking).

**Table 9:** Results of regression estimation (FMOLS and DOLS models).

Variables	FMOLS Model					DOLS Model				
	Individual Effects				Combined Effects (SDG)	Individual Effects				Combined Effects (SDG)
	SDG	SDG	SDG	SDG		SDG	SDG	SDG	SDG	
Constant	0.118∗∗	0.215∗∗	0.10∗	0.031∗	0.027∗∗	0.190∗∗	0.344∗	0.194∗∗	0.016∗	0.413∗∗
Δ***CTR***	***0.131∗∗***				***0.317∗∗***	***0.319∗***				***0.033∗∗***
Δ***PIT***		***0.073∗***			***0.203∗∗***		***0.103∗∗***			***0.189∗∗***
Δ***STR***			***0108∗***		***0.401∗***			***0.211∗∗***		***0.026∗∗∗***
Δ***ETR***				***0.090∗∗***	***0.211∗∗∗***				***0.300∗∗***	***0.083∗∗∗***
ΔGR	0.129∗	0.318∗	0.216∗∗	0.011∗∗	0.107∗∗	0.103∗∗	0.026∗	0.016∗	0.083∗	0.294∗∗
ΔINF	−0.038	−0.045	−0.031	−0.204	−0.084	−0.108∗	−0.003	−0.048∗	−0.118	−0.012∗
ΔFD	0.347∗	0.462∗∗	0.227∗	0.156∗∗	0.204∗	0.109	0.261∗	0.121∗∗	0.186	0.128∗
ΔFOP	0.056∗∗	0.069∗	0.008∗∗	0.401∗	0.088∗	0.318∗∗	0.155∗∗	0.270∗	0.217∗∗	0.135∗
ΔPSI	0.120∗	0.377∗∗	0.014∗	0.211∗∗	0.011∗	0.001∗∗	0.072∗	0.123∗∗	0.327∗	0.131∗∗
R-square	0.6672	0.6381	0.6471	0.6294	0.6907	0.4741	0.4441	0.4371	0.4829	0.4931
Adj. R-square	0.6563	0.6212	0.6261	0.6089	0.6817	0.4536	0.4179	0.4236	0.4658	0.4812
SE of regression	2.367	1.109	1.044	1.209	1.177	0.784	2.128	0.918	1.150	2.083
Long-run variance	0.002	0.010	0.007	0.028	0.019	0.016	0.047	0.051	0.016	0.011

∗∗∗, ∗∗, ∗ indicates 1%, 5%, and 10% level of significance, respectively.

Source: Authors constructed

## 6 Conclusion, policy implications, and future research scope

The study aims to examine the effects of the corporate tax rate on sustainable development in the BRIC and CIVETS countries. This research employs a panel dataset for 2000–2021 years and applies panel data regression model to analyse the data. The study confirms the results checking the robustness through the fully modified ordinary least square and the dynamic ordinary least square panel estimate methods. The study passes several tests like cross-sectional dependence tests, unit root tests, and model selection tests before conducting the focal part of the analysis. Corporate tax policies in BRIC and CIVETS countries bring about the necessary condition for economic progress, job creation, and poverty reduction. Thus, while no cure exists, it is clear that closer convergence of investment and tax policy is necessary to promote investment, job creation, and economic growth. International business continues to be a potent instrument for assisting people in escaping poverty. Taxation is inextricably related to development since it generates the funds required for states to mobilize resources and strengthen a country's infrastructure particularly in the emerging economies like BRIC and CIVETS.

The research finds that the corporate tax rate is positively and significantly associated with the sustainable development goals (SDG). The result implies that a higher rate of corporate tax plays vital role in achieving the sustainable development goals in the emerging economies. By including personal income tax, sales tax, and theoretical arguments, the study contributes to the debate on the corporate tax rate and SDG achievement in emerging countries. The study applies both individual effects and combined effects of corporate tax rate, personal income tax, sales tax, and effective tax rate with SDG. In both cases, the research finds significant and positive association of taxation with SDG. Thus, the research argues that achieveing the SDG of emerging economies depends on the countries' taxation rate and policy. This research employs the most updated data set that also contributes to the existing literature of emerging economies. Thus, the findings generated from this study can be a policy dialogue for the academics, policy-makers and government bodies of BRIC and CIVETS countries and other emerging economies as well.

Taxation is an important section of any country because the government can collect the maximum collection through taxation. Thus, changes in taxation have a great impact on the economy and the country's environment. This study finds that taxation through corporate tax, personal income tax, sales tax, and effective tax rate positively influences the emerging nations' sustainable development goals. Thus, the policymakers of the emerging economies should target the taxation policy in achieving SDGs. The policymakers can update the taxation policies through higher rates of corporate tax, individual personal tax, effective tax rate, and sales tax. There is no chance to avoid/lower tax collection if the nations want to achieve SDGs, thus, the decisionmakers should develop compulsory measures that ensure complete compliance of tax rules and regulations.

This research has some limitations: First, the study argues that the corporate tax, personal income tax, sales tax, and effective tax rate are the factors that enhance sustainable development goals. The findings are limited to the emerging nations, thus, the generalization issue may arise if the results are compared with developed or non-developed economies. Second, the sample size is only 220 because of country-specific data. Thus, future studies should collect industry-specific data and enlarge the sample size. Third, this study is fully designed on quantitative research, but taxation is very complex if there is a lack of qualitative data from the policymakers. Thus, future studies must collect the qualitative data and apply mixed method research. Finally, the future researchers should connect more variables like sustainable economic performance, green taxation, technological innovation, sustainable finance, etc. to make the study more interesting and wider reader coverage.

## Availability of data and material

Data are available at SDG Index^3^, Tax Foundation^4^, Trading Economics^5^, OECD Statistics^6^, WDI^7^, IMF^8^, KNOEMA^9^, and Global Economy^10^.

## Declarations

### Author contribution statement

MMR conceived and designed the experiments; performed the experiments; analyzed and interpreted the data; and wrote the paper.

### Funding statement

This research did not receive any specific grant from funding agencies in the public, commercial, or not-for-profit sectors.

### Data availability statement

Data will be made available on request.

### Declaration of interest’s statement

The authors declare no conflict of interest.

### Additional information

No additional information is available for this paper.

## Footnotes

1 Corporate Tax Rates Around The World, 2021–Tax Foundation: https://files.taxfoundation.org/20211207171421/Corporate-Tax-Rates-around-the-World-2021.pdf

2 https://www.oecd.org/ctp/tax-and-developing-countries.

3 https://dashboards.sdgindex.org/profiles.

4 https://taxfoundation.org/publications/corporate-tax-rates-around-the-world/.

5 https://tradingeconomics.com/.

6 https://stats.oecd.org/.

7 https://databank.worldbank.org/source/world-development-indicators.

8 https://www.imf.org/en/Data.

9 https://knoema.com/NBERCIFOI2017/the-chinn-ito-financial-openness-index.

10 https://www.theglobaleconomy.com/rankings/wb_political_stability/.
