# Corrigendum to “The effect of taxation on sustainable development goals: evidence from emerging countries” [Heliyon 8 (9), (September 2022) Article e10512]

**DOI:** 10.1016/j.heliyon.2022.e12279

**Published:** 2022-12-10

**Authors:** Md. Abdul Halim, Md. Mominur Rahman

**Affiliations:** a Department of Business Administration, Mawlana Bhashani Science and Technology University, Santosh, Tangail, 1902, Bangladesh; b Department of Business Administration, Institute of Science Trade and Technology, Dhaka, 1216, Bangladesh; c Department of Accounting & Information Systems, Comilla University, Cumilla, 3506, Bangladesh; d Department of Business Administration, Northern University Bangladesh, Dhaka, 1213, Bangladesh

In the original published version of this article, Md. Abdul Halim was not included as a co-author. This has now been corrected, with Md. Abdul Halim added as the first co-author. The corrected version of the Author Contribution Statement can be found below. The authors apologize for the errors. Both the HTML and PDF versions of the article have been updated to correct the errors.

**Author contribution statement:**

Md. Abdul Halim, Md. Mominur Rahman: Conceived and designed the experiments; Performed the experiments; Analyzed and interpreted the data; Contributed reagents, materials, analysis tools or data; Wrote the paper.

**Declaration of interest's statement:**

The authors declare no conflict of interest.

